# MRI characteristics and oncological follow-up of patients with ISUP grade group 4 or 5 prostate cancer

**DOI:** 10.1007/s00261-023-04073-y

**Published:** 2023-10-31

**Authors:** M. Boschheidgen, L. Schimmöller, R. Kastl, L. R. Drewes, K. Jannusch, K. L. Radke, J. Kirchner, T. Ullrich, G. Niegisch, P. Albers, G. Antoch, J. P. Radtke

**Affiliations:** 1 Department of Diagnostic and Interventional Radiology, Medical Faculty, University Dusseldorf, Moorenstr. 5, 40225 Dusseldorf, Germany; 2 Department of Urology, Medical Faculty, University Dusseldorf, Moorenstr. 5, 40225 Dusseldorf, Germany; 3 Department of Diagnostic, Interventional Radiology and Nuclear Medicine, Marien Hospital Herne, University Hospital of the Ruhr-University Bochum, Herne, Germany

**Keywords:** Prostatic neoplasms, Multiparametric magnetic resonance imaging, Risk stratification, High-risk prostate cancer

## Abstract

**Objectives:**

To analyze multiparametric MRI (mpMRI) characteristics of patients with International Society of Urological Pathology (ISUP) grade group (GG) 4 or 5 prostate cancer (PC) and to correlate MRI parameters with the occurrence of biochemical recurrence (BCR) after radical prostatectomy (RPE).

**Methods:**

In this single-center cohort study consecutive patients with mpMRI and ISUP GG 4 or 5 PC were retrospectively analyzed. Clinical, MR-guided biopsy, and diagnostic mpMRI parameter were assessed. A subcohort of patients with RPE and follow-up was analyzed separately. A univariate and multivariate analyses were performed to determine parameters that are associated to patients with BCR after RPE.

**Results:**

145 patients (mean age 70y, median PSA 10.9 ng/ml) were analyzed. 99% had a PI-RADS classification of 4 or 5, 48% revealed MRI T3 stage, and median diameter of the MRI index lesion (IL) was 15 mm. IL showed a median ADC value of 668 ×10^−6^ mm^2^/s and exhibited contrast enhancement in 94% of the cases. For patients with follow-up after RPE (*n* = 82; mean follow-up time 68 ± 27 m), MRI parameters were significantly different for contact length of the IL to the pseudocapsule (LCC), MRI T3 stage, and IL localization (*p* < 0.05). Higher PSAD and MRI T3 stage were independent parameters for the risk of BCR when incorporating clinical, biopsy, and MRI parameters.

**Conclusion:**

ISUP GG 4 or 5 PC has distinctive characteristics on mpMRI and were detected on MRI in all cases. In addition, higher PSAD and MRI T3 stage were significant predictors for BCR after RPE.

**Supplementary Information:**

The online version contains supplementary material available at 10.1007/s00261-023-04073-y.

## Introduction

Locally confined prostate cancer (PC) is prognostically stratified as low, intermediate, or high risk [1–3]. There are several therapeutic approaches for newly diagnosed PC which includes active surveillance, radical prostatectomy (RPE) (with/without pelvic lymph node dissection), and radiotherapy (RT) (with/without androgen deprivation therapy). High-risk PC causes most of the cases of cancer-specific death [4]. Following European Association of Urology (EAU), high-risk PC is defined as Gleason score ≥ 8 (International Society of Urological Pathology grade group; ISUP GG ≥ 4), prostate-specific antigen (PSA) >20 ng/ml, or clinical stage ≥T2c, but this definition varies between different guidelines.

Multiparametric magnetic resonance imaging (mpMRI) already has an important role in the diagnostic pathway, local tumor staging, and for predicting the presence of lymph node metastasis. Moreover, it contains information about the aggressiveness and might be useful in cancer grading [5–7]. To date, calculation of the risk for recurrence of PC is still based on clinical parameters only, for example, as assessed by the EAU risk group classification. These parameters include the clinical T stage, PSA level, and tumor’s Gleason grade group [3]. Several studies already revealed that the combination of clinical parameters and parameters extracted from pre-biopsy mpMRI may improve the performance of risk stratification for distant metastasis and biochemical recurrence (BCR) [8–15]. Mazzone et al. developed a novel classification integrating clinical and MRI parameters to assess the risk for disease recurrence. As this classification offers higher accuracy compared to conventional D’Amico risk stratification, it could have an impact on future clinical decision-making in terms of treatment strategy and clinical follow-up [15]. However, MRI-based risk stratification is still not incorporated in the clinical decision-making process so far.

The aim of this study is the evaluation of MRI parameters in patients with high-grade PC and to determine parameters which may identify patients facing a higher risk for BCR. This knowledge could contribute to a more individualized way of therapeutic planning and follow-ups.

## Materials and methods

**Fig. 1:**
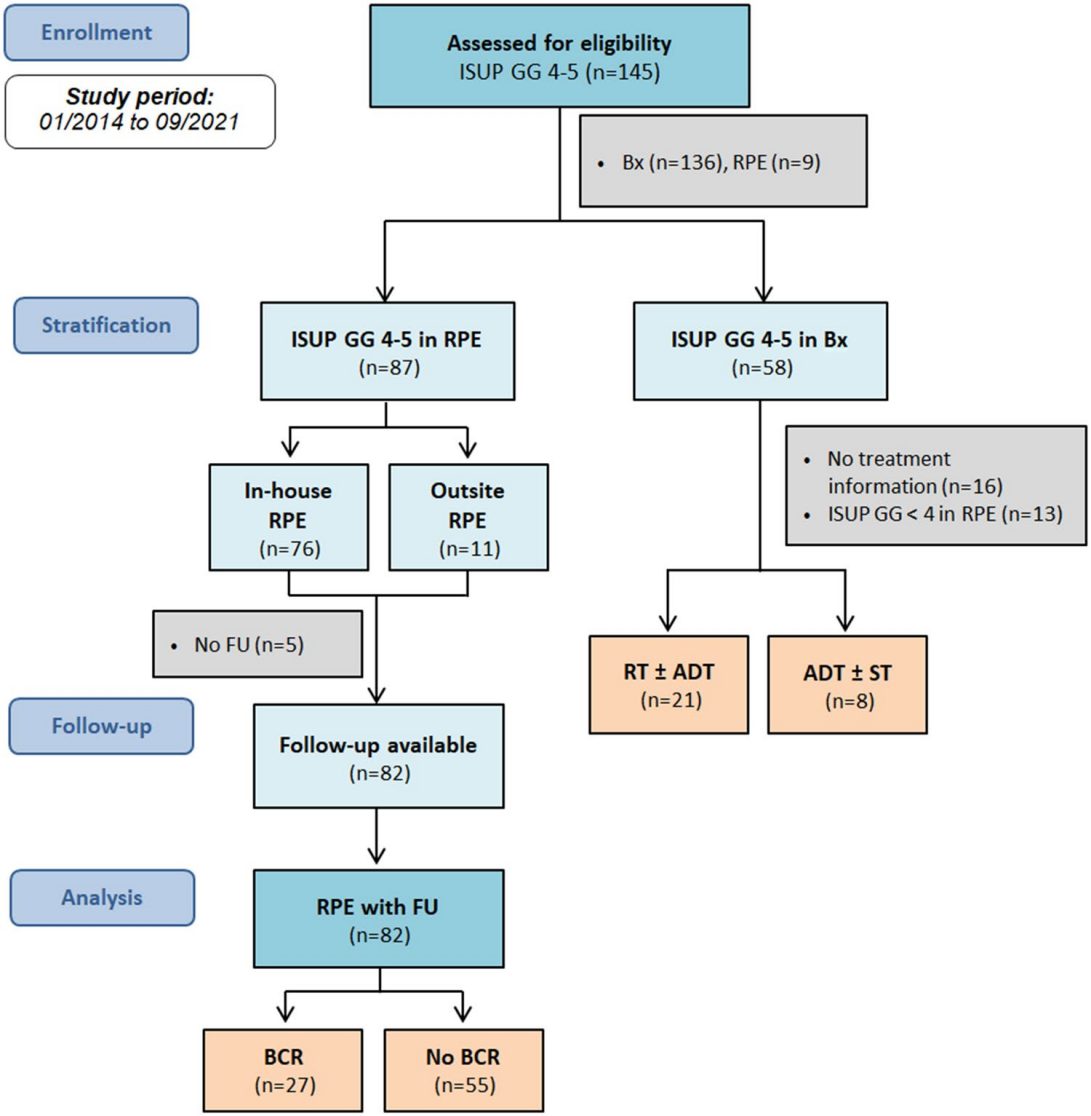
Flowchart of study design and patient selection. *ISUP* International Society of Urological Pathology, *GG* grade group, *RPE* radical prostatectomy, *BX* biopsy, *RT* radiation therapy, *ADT* androgen deprivation therapy, *ST* systematic therapy, *FU* follow-up, *BCR* biochemical recurrence

The study was approved by local ethics committee (Medical Faculty of the Heinrich Heine University Düsseldorf; Study-Nr: 5910R). Written informed consent was obtained from every patient. Consecutive patients with mpMRI and the diagnosis of ISUP GG 4 and 5 PC between January 2014 and September 2021 were enrolled. The classification as ISUP GG 4 or 5 referred either to the histopathology from targeted or systematic biopsy or from the radical prostatectomy specimen, if available (Fig. 1). The patient was included even if the other test showed a lower subgroup. All patients received mpMRI of the prostate at our institution. Subsequently, targeted MRI/ultrasound fusion-guided biopsy combined with systematic 12-core transrectal ultrasound-guided prostate biopsy was conducted. Inclusion criteria were treatment naïve high-grade PC diagnosed in our center and available data for MRI and biopsy. Clinical and biopsy information contained age, PSA, PSAD, ISUP GG, and percentage of PC infiltration per core. MRI parameters included PI-RADS v2.1 classification, localization of the index lesion (IL) (peripheral zone, PZ or transitional zone, TZ), largest diameter, contact length to prostatic pseudocapsule (LCC), MRI T stage, ADC value, and focal DCE positivity of the IL. All patients were primarily treated either with RPE (with/without pelvic lymph node dissection), RT (with/without androgen deprivation therapy), or systematic therapy for metastatic disease. Follow-up post-treatment included periodical PSA testing every three months. As a population of special interest, we analyzed those patients, who were treated with RPE focusing on the occurrence of BCR. All patients with RPE had staging with computed tomography of thorax, abdomen, and pelvis to exclude distant metastasis at diagnosis, so localized high-grade PC was the inclusion criteria for this subgroup. Lymphogenic metastasis was defined as lymph nodes with a short axis diameter of more than one centimeter on computed tomography. BCR was defined as an increase in serum PSA levels above 0.2 ng/ml [16]. The study objective was to identify parameters, which characterize high-grade PC in mpMRI and to correlate MRI parameters with BCR at follow-up after RPE.

### Imaging acquisition

All mpMRI scans were conducted on 3T MRI scanners using either an 18-channel phased-array surface coil combined with a 32-channel spine coil or a 60-channel phased-array surface coil. MRI parameters were in line with the PI-RADS version 2.1 and contained T2-weighted turbo spin echo (TSE) sequences in three planes (T2WI; axial: voxel size 0.5 × 0.5 × 3.0 mm; FOV 130 mm), diffusion-weighted imaging [DWI; z-EPI (ZOOMit, Siemens Healthineers) and rs-EPI (RESOLVE, Siemens Healthineers); voxel size 1.4 × 1.4 × 3.0 mm; *b*-values 0, 500, 1000 s/mm^2^ plus calculated 1800 s/mm^2^], and dynamic contrast-enhanced imaging (DCE; T1 vibe; voxel size 0.8–1.5 × 0.8– 1.5 × 3.0 mm, scan time 3 min, temporal resolution 7 sec) [17]. Apparent diffusion coefficient (ADC) parameter maps were calculated by the scanner using the standard monoexponential model.

### Biopsy and pathology

Targeted and systematic 12-core biopsy were conducted on an MRI/US fusion-guided biopsy system with elastic registration (UroNAV, Invivo) using an 18G fully automatic biopsy gun (Bard Medical) by experienced urologists with over 5 years of experience. Two targeted cores were taken from each suspicious lesion on mpMRI. Biopsies were evaluated according to the ISUP 2014 classification. For evaluation of RPE, ISUP GG, and extraprostatic extension (T3a/T3b), margin status (R1) and lymph node metastases (N1) were analyzed. In case of discrepant findings in histopathology of biopsy and RPE, the results from RPE specimen were considered as gold standard.

### Image analysis

MpMRI data were retrospectively evaluated by two readers in consensus (M.B. and L.S.) with 4 and 11 years of experience in reading prostate MRI. Prostate volume was measured by software volumetric analysis (DynaCAD, Philips Healthcare) and PSA density (PSAD) was calculated by dividing PSA blood levels by prostate volume. Images were scored according to PI-RADS v2.1. PC localization, maximum diameter, and LCC were defined by the correlating IL measured in T2w sequences on the slice with the largest diameter. MRI T staging was performed. EPE or seminal vesicle infiltration was present if PC crossed the prostate pseudocapsule (≥ 3 mm) (cT3a) or extended continuously into the seminal vesicles (cT3b). ADC values were measured by placing a circular region of interest into the IL. DCE was scored as positive if the IL showed a focal, earlier, or contemporaneous with enhancement of adjacent normal prostatic tissues and corresponds to a suspicious finding on T2W and/or DWI following the criteria of PI-RADS v2.1.

### Statistical analysis

Statistics were performed using IBM SPSS® Statistics (Version 29, IBM Corp). *p*-values < 0.05 were defined as statistically significant. Wilcoxon signed rank test was performed to compare continuous data; chi square test was performed to compare categorical data. For analysis of the subcohort with RPE and follow-up, we conducted a univariate and multivariate logistic regression analyses to check for significant parameters to have an influence on BCR. We conceived three different combinations of parameters, the first with clinical parameter only, the second with clinical plus biopsy parameter, and a third model with clinical plus biopsy plus MRI parameter.

## Results

### Study population

145 patients with histologically proven ISUP 4 or 5 PC [mean age 70 ± 8 y; median PSA 10.9 ng/ml (IQR 6.8–19.1 ng/ml); median PSAD 0.25 ng/ml/cm^3^ (IQR 0.16–0.42 ng/ml/cm^3^)] in either biopsy at baseline or RPE specimen were finally included. Follow-up data were available for 120 of 145 patients with follow-up period of 68 ± 27 months. PC-related death was the case in 7 patients (6%). Regarding the tumor therapy, we observed different strategies in our collective, although the majority was treated with robot-assisted RPE which was the case in 100 of 145 patients. 21 patients were treated with RT of the prostate with/without hormone therapy (ADT). Eight patients were treated with systemic treatment (ST) due to metastatic disease at diagnosis. In 16 patients, information about therapeutic decision after diagnosis was not available. A CONSORT flowchart of the study population is shown in Fig. 1.

### Clinical and MRI characteristics

**Fig. 2:**
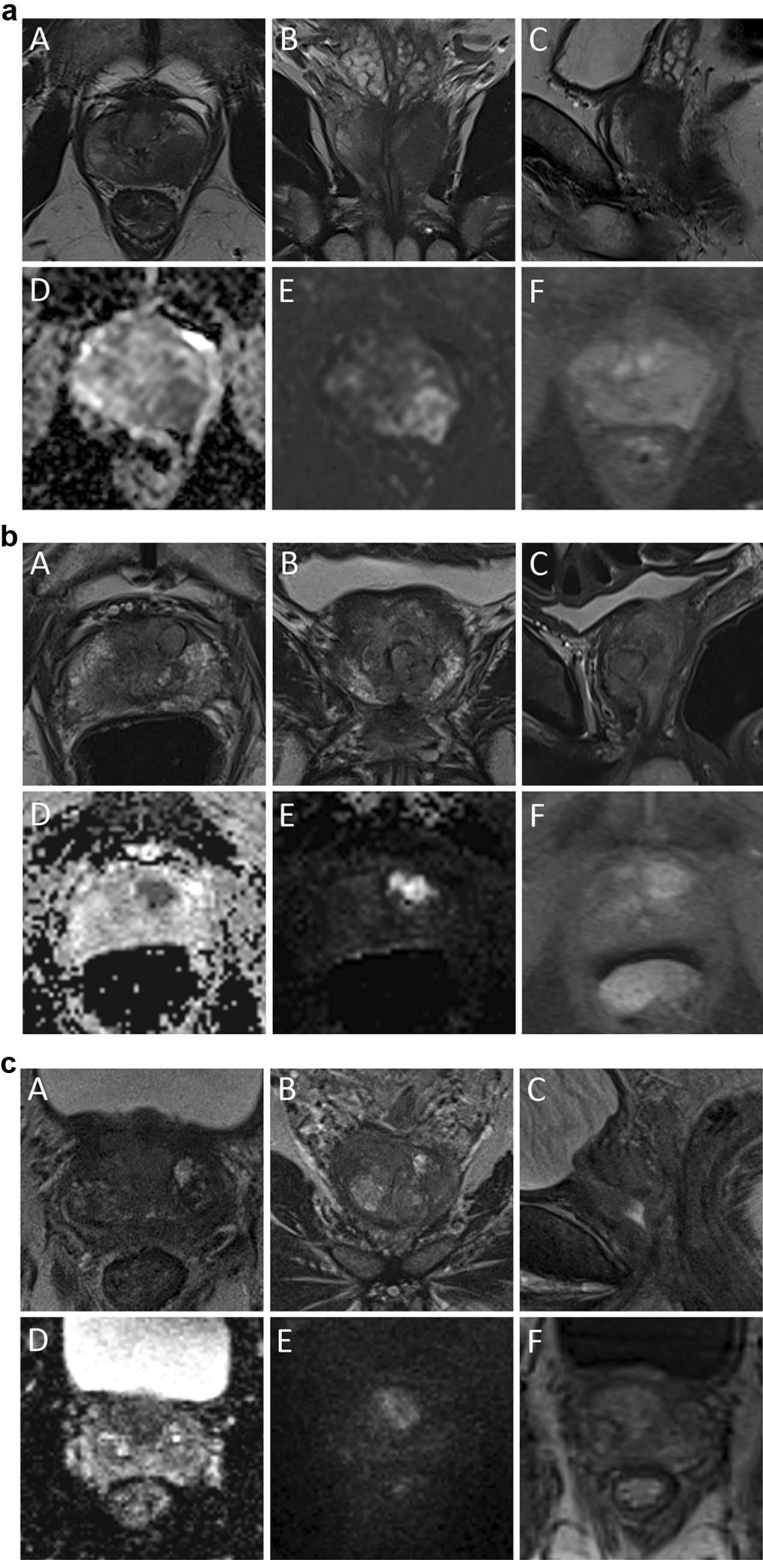
**a** 52 years, PSA 1.3 ng/ml, PSAD 0.03 ng/ml/cm^3^; Gleason 4+4 = 8 (ISUP GG 4) with 50% infiltration in biopsy cores; and pT3 pN0 R1 with Gleason 4+4 = 8 (ISUP GG 4) at RPE histopathology. A–C: T2w images, D, E: DWI, F: DCE; index lesion in left PZpl with MRI cT3 stage. **b** 78 years, PSA 14.2 ng/ml, PSAD 0.38 ng/ml/cm^3^; maximum tumor diameter 10 mm in MRI; Gleason 4+5=9 (ISUP GG 5) with 75% infiltration in biopsy cores; and recurrence after RT 2 years. A–C: T2w images, D, E: DWI, F: DCE; index lesion in anterior TZ with MRI cT2c stage. **c** 65 years, PSA 9.85 ng/ml, PSAD 0.20 ng/ml/cm^3^; Gleason 3+3 = 6 (ISUP GG 1) with 10% infiltration in biopsy cores; and pT2 pN0 R0 with Gleason 4+5 = 9 (ISUP GG 5) at RPE histopathology. A–C: T2w images, D, E: DWI, F: DCE; index lesion in anterior TZ with MRI cT2c stage

Clinical and MRI characteristics of all patients are shown in Table 1. PSA values were reaching from 1.27 to 80.4 ng/ml, 8 patients had a PSA ≤ 4.0 ng/ml at biopsy (6%) **(**Fig. 2a**)** and 31 revealed a PSAD < 0.15 ng/ml/ml (21%). Patients with PSA ≤ 4.0 ng/ml were referred to MRI either due to clinical suspicion for PC (e.g., digital rectal examination, *n* = 6 or positive family history, *n* = 2). All patients had a PI-RADS v2.1 classification ≥ 3 and 99% revealed a PI-RADS ≥ 4. PC IL diameter at baseline MRI ranged from 4 to 43 mm (Fig. 2b). MRI showed extracapsular extension or seminal vesicle infiltration in 70 of 145 patients and tumor exhibited contact to prostatic pseudocapsule in 95% in our cohort. Focusing on diffusion-weighted imaging, 8 patients had ADC values above 1000×10^−6^ mm^2^/s, 88 patients had ADC values between 600 and 1000×10^−6^ mm^2^/s, and 49 patients had ADC values below 600x10^−6^ mm^2^/s. We observed focal enhancement of PC IL in 94%.

**Table 1:** Clinical, MR-guided biopsy, and MRI parameters at baseline

	All patients	RPE subcohort
Patients (*n*)	145	82
Age in years; median (IQR)	71 (65–76)	68 (65–73)
PSA in ng/ml; median (IQR)	10.9 (6.8–19.1)	9.6 (6.5–17.8)
PSAD median (IQR)	0.25 (0.16–0.42)	0.23 (0.14–0.39)
ISUP GG after MR-guided biopsy(n)		
1	1*	1*
2	4*	4*
3	4*	4*
4	82	41
5	55	32
Percentage of infiltration in biopsy core median (IQR)	70 (50–85)	65 (50–80)
PI-RADS v2.1 (*n*)		
3	2	2
4	43	22
5	100	58
T2		
IL localization		
PZ	112	67
TZ	33	15
IL diameter in mm; median (IQR)	15 (12–22)	17 (12–22)
Contact length to pseudocapsule (LCC) in mm; median (IQR)	17 (12–26)	18 (12–26)
MRI T3 stage in %	48	51
DWI		
ADC value of IL in ×10^−6^ mm^2^/s. median (IQR)	668 (571–798)	662 (544–785)
DCE		
IL with focal enhancement on DCE in %	94	96

*MRI* magnetic resonance imaging, *PSA* prostate specific antigen, *PSAD* prostate specific antigen density, *TZ* transition zone, *PZ* peripheral zone, *DWI* diffusion weighted imaging, *ADC* apparent diffusion coefficient, *DCE* dynamic contrast enhancement, *IQR* interquartile range, *IL* index lesion; *T stage* Tumor stage, *ISUP* International Society of Urological Pathology, *GG* grade group, *RPE* radical prostatectomy

*ISUP GG 4 or 5 in RPE

Considering MRI/US fusion-guided biopsy data, only targeted biopsy revealed the PC in 25 patients and targeted biopsy revealed a higher ISUP grade group PC than the systematic biopsy in 36 patients. In 7 patients, only the systematic biopsy diagnosed the presence of PC. Mean percentage of infiltration in biopsy cores was higher for targeted biopsy than for systematic biopsy (56 ± 31% vs. 39 ± 31%). The results of targeted and systematic biopsy are shown in Supplemental Table 1.

### Subcohort with RPE and follow-up

At histopathology of RPE specimen, 9 patients showed an upgrading of the ISUP GG compared to the biopsy histopathology (*n* = 7 to ISUP GG 5; *n* = 2 to ISUP GG 4, Fig. 2c), while a downgrading was observed in 18 patients (*n* = 5 with ISUP GG 5 to ISUP GG 4; *n* = 13 to ISUP GG ≤ 3). Only moderate correlation between histopathology at biopsy and RPE was observed (Spearman *ρ* = 0.41; *p*<0.001). Finally, 87 patients revealed ISUP GG 4 or 5 cancer in histopathology of RPE specimen, 82 of them with follow-up data available (Fig. 1). In this subcohort RPE showed pT2a stage in 10 patients, pT2c in 17 patients, pT3a in 22, and pT3b in 33 patients. Regarding T3 stage, concordance of MRI and pathology was 77%. 17 patients (21%) had lymph node metastasis and 31 patients (38%; *n* = 27 with pT3b; *n* = 4 with pT2) had positive margins (R1). Median follow-up period of this subgroup was 78 months (IQR 57–89 months). BCR was observed in 27 of 82 patients (33%) with a median time interval after diagnosis of 32 months (IQR 21-51 months).

**Fig. 3:**
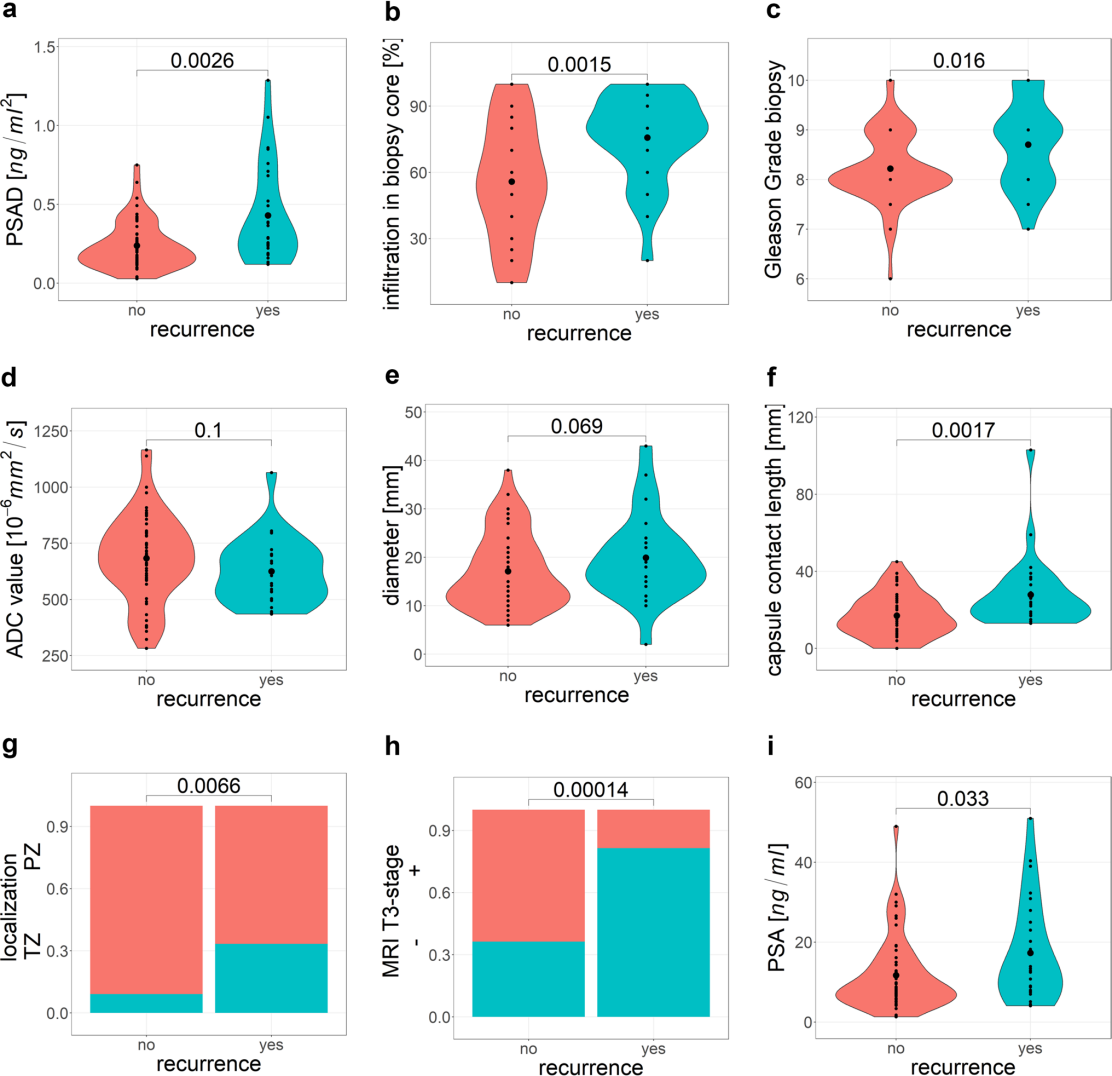
Violin plots for the distribution of determined parameters for patients with disease recurrence and cancer-free patients

Comparing patients with and without BCR, we detected higher PSA with 17.3 ng/ml vs. 11.7 ng/ml (*p* = 0.033) and higher PSAD with a mean of 0.43 ng/ml/ml vs. 0.24 ng/ml/ml (*p* = 0.003). Considering biopsy parameters, percentage of infiltration in biopsy cores differed significantly (median infiltration 60%, IQR 30–80% vs. 40%, IQR 10–60%; *p* = 0.002). Regarding imaging parameters measured on MRI, significant differences were observed for LCC (median 22 mm vs. 15 mm, *p* = 0.002), MRI T3 stage (81% vs. 36%, *p* < 0.001), and tumor localization (TZ 33% vs. 18%, *p* = 0.015). Violin plot diagrams are illustrated in Fig. 3 to visualize the distribution of the data. For the data extracted from histopathology of RPE, 16 of 27 patients with BCR had R1, while 15 of 55 patients without BCR exhibited R1 postoperatively (*p* < 0.01).

Univariate analysis is shown in Table 2. In the multivariate regression analysis consisting of clinical parameters only (*p* = 0.33), PSAD was an independent predictor for BCR (*p* = 0.03) (Table 3). When extending the analysis with MRI-guided biopsy parameters (*p* = 0.34), PSAD and infiltration in biopsy core were significantly associated with recurrence (*p* < 0.05). In the third combination with clinical plus biopsy plus MRI parameters (*p* = 0.015), which exhibited significant relation with BCR at univariate analysis, T3 stage at MRI (*p* = 0.05) and PSAD (*p* = 0.03) where significant parameters to predict BCR at follow-up.

**Table 2:** Univariate analysis of different parameters and their influence on BCR

*n* = 82	*B*	Std. Error	95%CI	*p*
Clinical parameter				
Age	− 0.06	0.04	− 1.45–8.95	0.101
** PSA**	**0.05**	**0.02**	**0.01–0.10**	**0.032**
** PSAD**	**3.93**	**1.29**	**1.63–6.74**	**0.002**
Biopsy parameter				
** ISUP GG**	**0.85**	**0.33**	**0.22–1.54**	**0.011**
** Infiltration**	**0.03**	**0.01**	**0.01–0.06**	**0.002**
MRI parameter				
**Localization**	**1.61**	**0.62**	**0.42–2.90**	**0.010**
Diameter	0.04	0.03	− 0.01–0.10	0.142
ADC value	− 0.002	0.001	− 0.01–0.01	0.170
** MRI T stage**	**2.04**	**0.57**	**0.99–3.26**	**<0.001**
** LCC**	**0.06**	**0.02**	**0.02–0.11**	**0.005**

*MRI* magnetic resonance imaging, *PSA* prostate-specific antigen, *PSAD* prostate-specific antigen density, *ISUP* International Society of Urological Pathology, *GG* grade group, *ADC* apparent diffusion, *T stage* Tumor stage, *LCC* length to prostatic pseudocapsule, *B* estimation coefficient, *Std. Error* standard error, *CI* confidence interval, *p* p-value

*p*-values <0.05 are given as statistically significant

**Table 3:** Multivariate logistic regression analysis of BCR after RPE

*n* = 82	*B*	Std. Error	95%CI	*p*
Clinical parameter				
Age	− 0.070	− 0.043	− 0.16 to 0.01	0.11
PSA	0.0004	0.004	− 0.08 to 0.07	0.99
** PSAD**	**3.99**	**1.85**	**0.70 to 8.12**	**0.03**
Clinical and biopsy parameter				
Age	− 0.09	0.05	− 0.19 to 0.001	0.06
PSA	− 0.01	0.05	− 0.12 to 0.07	0.81
** PSAD**	**4.03**	**2.13**	**0.34 to 8.84**	**0.05**
** ISUP GG**	**0.74**	**0.40**	− **0.02 to 1.55**	**0.04**
** Infiltration**	**0.03**	**0.01**	**0.01 to 0.05**	**0.02**
Clinical, biopsy, and MRI parameter				
PSA	− 0.07	0.05	− 0.19 to 0.03	0.217
** PSAD**	**5.69**	**2.65**	**1.03 to 11.47**	**0.032**
ISUP GG	0.47	0.41	− 0.32 to 1.30	0.25
Infiltration	0.02	0.01	− 0.01 to 0.04	0.11
Localization	0.93	0.84	− 0.72 to 2.65	0.27
** MRI T stage**	**1.43**	**0.74**	**0.02 to 0.09**	**0.05**
LCC	0.02	0.03	− 0.02 to 0.09	0.37

*MRI* magnetic resonance imaging, *PSA* prostate-specific antigen, *PSAD* prostate-specific antigen density, *ISUP* International Society of Urological Pathology, *GG* grade group, *LCC* length to prostatic pseudocapsule, *IL* index lesion, *T stage* Tumor stage, *BCR* biochemical recurrence, *RPE* radical prostatectomy, *B* unstandardized coefficient, *Std. Error* standard error, *CI* confidence interval, *p* p-value

*p*-values <0.05 are given as statistically significant

## Discussion

To our knowledge, this is the first study to report on MRI parameters in a collective incorporating only patients harboring PC of ISUP GG 4 and 5. MRI was highly sensitive in detecting these high-grade PC with specific MRI characteristics like almost entirely PI-RADS 4 and 5 classification, primarily high lesion diameter and LCC, low ADC value, and focal enhancement on DCE. A combination of clinical, biopsy, and MRI parameters had the best discrimination between men suffering from BCR in follow-up as compared to clinical and/or biopsy parameters only.

When focusing on the PSA values of our study population, it is notable that 8 patient revealed a PSA below 4 ng/ml. Previously published results showed the reduced sensitivity of PSA as a reliable predictor for both the diagnosis and the risk stratification for disease recurrence [10, 15, 18]. Furthermore, we observed a relevant number of patients with PSAD below 0.15 ng/ml/ml. Although, PSAD has been described as an additional decision guidance in indeterminate cases and equivocal lesions, it is not always trustworthy due to prostate volume enlargement (especially in patients with benign prostate hyperplasia) [19]. To our experience, it may especially help in patients with smaller prostate volumes as a supportive tool for referring patients to biopsy. On the other side, we included 80 of 145 patients with a PSA value above 10 ng/ml. None of them were younger than 55 years (mean age 71 years). MRI showed T3 stage with extracapsular extension in 48 of 80 cases, so PC was already advanced at diagnosis in most of the patients. If screening and PSA control would have been conducted earlier in these patients, PC might be detected in a less advanced stage. As there is an ongoing discussion about a commonly accepted PC screening strategy, our results reinforce the role of mpMRI.

Moreover, ISUP GG (Gleason Grade, respectively) in biopsy seems to be an important parameter to predict recurrence, a fact that we could demonstrate in our model as well [20]. Interestingly, we observed a relevant number of PC only detected in targeted biopsy and furthermore 36 patients with higher ISUP GG at targeted biopsy than in the systematic cores, which underlines the important role of pre-biopsy MRI in the high-risk cohort as well. Although PC tends to be larger at this stage, there is still a risk of missing the tumorous areas in case of performing systematic biopsy alone. However, the correct ISUP grade of MRI-targeted biopsy is a fact that has been discussed controversy, as a previous study reported downgrading of Gleason Grade at subsequent histopathology after RPE [21, 22]. We observed similar values for upgrading and downgrading after surgery. What is more important are the cases, where histopathology of biopsy cores underestimated the tumors ISUP GG. These are cases where MRI can contribute and may play a key role for clinical decision-making. As these tumors can clearly be depicted and appear aggressive on MRI, clinicians should be warned if biopsy reveals only low- or intermediate risk cancers. As MRI can reliably grade cancer aggressive, re-biopsy should be conducted to minimize the risk of missing high-grade PC [5].

Comparing patients with BCR at follow-up and cancer-free survival after RPE, we observed remarkable differences not only in clinical parameters and RPE histopathology, but also in defined MRI parameters. Our data support recent evidence that data derived from MRI can contribute to identify patients with higher and lower risk for BCR and may play a role to predict recurrence [14, 15, 21, 23]. Although, all our patients would be categorized as high risk (following EAU guidelines) based on their ISUP GG, the data suggest discrepancies at baseline which might have an influence on prognostic outcome. The correlation between ADC values and PC aggressiveness has been described earlier [7, 24].

Moreover, MRI has the ability to predict extracapsular extension and cT stage and is a valid predictor in PC staging [25, 26]. For non-metastatic disease in the high-risk group, surgical treatment remains the first option and adjuvant treatment (e.g., radiation therapy or androgen deprivation) might be necessary in some cases, based on the results of RPE histopathology.

In our multivariate logistic regression analysis incorporating information from PSA and biopsy results, PSAD and infiltration in biopsy core were independent parameters to estimate the risk of disease recurrence. These findings are not surprising and in accordance with previous studies showing the important role of Gleason grade [26]. When including MRI parameters into the multivariate regression analysis, the MRI T3 stage and PSAD correlated significantly with the risk for BCR. This is consistent with published data from pathology studies, showing an association between BCR and pathological T stage, pathological Gleason grade, and positive surgical margins [27]. As previously mentioned, extracapsular extension can be determined on MRI, and we observed a good correlation of MRI T stage with the histopathology of RPE [26]. A possible explanation for MRI T3 stage being the only independent MRI parameter could be a very high collinearity with maximum lesion diameter and LCC, which could mask their effect in the proposed analysis. There is already evidence in the literature about the benefit of MRI parameters in several prediction models [15, 28, 29]. However, these studies did not investigate on patients with a high-risk constellation only. As these groups of patients face higher risks for metastasis and prostate cancer-related death, a detailed knowledge about individual risk is helpful in clinical settings.

Some limitations of this study, besides the retrospective, single-center design, need to be discussed. First, mpMRI images were rated by individual radiologists. The rating might be influenced by experience, local in-house standards, and personal preference. This could probably bias the diagnostic performance of mpMRI. Second, we did not incorporate the information from RPE histopathology, as this study focused on the additional use of MRI in a high-risk collective at diagnosis. This means that we included patients for the analysis regardless of their R1-status. R1-status is known to have an influence on the risk for BCR and may act as a confounder. RPE histopathology contains important knowledge about cancer aggressiveness and can predict BCR at follow-up. Finally, at follow-up and subgroup analysis, we focused on patients treated with RPE only.

In conclusion, all high-grade PC could be clearly identified on mpMRI. PI-RADS classification was 5 in over 50%, lesion diameter and LCC tend to be higher than 12 mm, ADC was below 800 × 10^−6^ mm^2^/s, and DCE showed focal enhancement in over 90%. Higher MRI staging parameters next to higher PSAD seems to be associated to higher risk of BCR at follow-up and so may help clinicians to earlier identify these patients. However, the detection of advanced PC stages in older men might be reduced by standardized PSA screening incorporating MRI.

### Supplementary Information

Below is the link to the electronic supplementary material.

Supplementary file1 (DOCX 15 KB)

## Data Availability

The data that support the findings of this study are available from the corresponding author, Lars Schimmöller, upon reasonable request.

## Abbreviations

PC: Prostate cancer
RPE: Radical prostatectomy
RT: Radiotherapy
ISUP: International society of urological pathology
GG: Grade group
mpMRI: Multiparametric magnetic resonance imaging
EAU: European association of urology
PSA: Prostate-specific antigen
BCR: Biochemical recurrence
TB: Targeted biopsy
SB: Systematic biopsy
PSAD: Prostate-specific antigen density
PZ: Peripheral zone
TZ: Transition zone
LCC: Length of capsule contact
ADC: Apparent diffusion coefficient
DCE: Dynamic contrast enhancement
DWI: Diffusion-weighted imaging
EPE: Extraprostatic extension
IL: Index lesion
PI-RADS: Prostate imaging and reporting archiving data system
AUC: Area under the curve
ROC: Receiver operator characteristics
CI: Confidence interval
p: *p*-Value
