# Supplementary material for: MRI characteristics and oncological follow-up of patients with ISUP grade group 4 or 5 prostate cancer
Source: Abdom Radiol (NY). 2023 Oct 31;49(1):192–201. doi: 10.1007/s00261-023-04073-y (PMC10789849; doi:10.1007/s00261-023-04073-y)
Supplement: Supplementary file 1 — Supplementary file1 (DOCX 15 KB) [file 261_2023_4073_MOESM1_ESM.docx]

**Supplemental Table 1:** Distribution of ISUP grade group (ISUP GG) at MRI-guided biopsy and systematic biopsy.

|  | | **Targeted biopsy (TB)** | | | | | | **All** |
| --- | --- | --- | --- | --- | --- | --- | --- | --- |
|  |  | N | **1** | **2** | **3** | **4** | **5** |  |
| **Systematic biopsy (SB)** | N | 0 | 0 | 2 | 2 | 14 | 7 | 25 |
|  | **1** | 0 | 1 | 0 | 0 | 4 | 1 | 6 |
|  | **2** | 0 | 0 | 2 | 0 | 11 | 2 | 15 |
|  | **3** | 1 | 0 | 0 | 1 | 8 | 3 | 13 |
|  | **4** | 4 | 0 | 7 | 8 | 25 | 7 | 51 |
|  | **5** | 2 | 0 | 1 | 1 | 6 | 25 | 35 |
| **All** | | 7 | 1 | 12 | 12 | 68 | 45 | 145 |

*N = negative*
